# A Multi-Locus and Machine Learning-Based Assessment of SNCA Variants in Alzheimer’s Disease

**DOI:** 10.3390/ijms27115143

**Published:** 2026-06-05

**Authors:** Hatice Segmen, Mustafa Yildiz

**Affiliations:** 1 Department of Neurology, Kanuni Sultan Suleyman Training and Research Hospital, Saglik Bilimleri University, 34303 Istanbul, Türkiye; haticesegmen@hotmail.com; 2 Faculty of Medicine, Department of Biophysics, Trakya University, 22030 Edirne, Türkiye

**Keywords:** Alzheimer’s disease, alpha-synuclein, *SNCA* gene, rs2583988, rs2619363, rs2619364, rs10005233

## Abstract

This study investigates the role of single nucleotide polymorphisms (SNPs) in the *SNCA* gene, encoding alpha-synuclein, in Alzheimer’s disease (AD). A case–control study was conducted including 95 AD patients and 97 healthy controls. Four *SNCA* polymorphisms (rs2583988, rs2619363, rs2619364, rs10005233) were analyzed using logistic regression, haplotype estimation, genotype combination analysis, and Random Forest modeling. Significant associations were identified for rs2583988, rs2619364, and rs2619363, while rs10005233 showed no association. The rs2583988 C allele and rs2619364 G allele were more frequent in patients, suggesting increased disease risk. Linkage disequilibrium analysis revealed weak correlations (low r^2^), indicating largely independent genetic effects. Multivariate logistic regression showed that clinical parameters, rather than genetic variants, were independently associated with AD. Multi-locus genotype analysis demonstrated that specific SNP combinations were linked to increased disease risk. Firth regression confirmed associations in low-frequency genotypes. The outcomes derived from the Random Forest methodology were classified as exploratory and not as proof of clinical predictive utility, attributed to the limited sample size, the absence of external validation, and the educational imbalance. Ordinal logistic regression indicated no association between *SNCA* variants and cognitive severity, while education had a protective effect. The selected *SNCA* variants showed exploratory associations with AD in this cohort; however, they failed to maintain their validity as independent predictors in multivariate logistic regression analysis. Before drawing any conclusions regarding screening or risk stratification, these findings require independent replication, correction for multiple testing and functional validation.

## 1. Introduction

Alzheimer’s disease (AD) is the most common neurodegenerative disease, leading to a progressive decline in memory, language, problem-solving, and other cognitive functions, and ultimately to dementia and death [1]. It is generally classified as early-onset (before 65 years of age) and late-onset (65 years of age and older); the late-onset group accounts for 94% of all AD cases [2]. Both genetic and environmental factors have been reported to play a role in the development of AD. To date, many genes associated with AD have been identified [3].

AD is neuropathologically characterized by brain atrophy and the accumulation of intraneuronal tangles composed of hyperphosphorylated forms of microtubule associated with protein tau (MAPT) and extracellular amyloid-β peptide plaques [4,5,6]. Genome-wide association studies (GWASs) have found that the MAPT gene, which encodes the MAPT protein, is associated with Parkinson’s disease (PD) [7,8,9]. Clinically, dementia often accompanies advanced PD and parkinsonian features can be seen in AD [10]. These overlapping features suggest a potential link between AD and PD.

Many clinical and pathological features are shared between two neurodegenerative diseases. Due to the toxicity of α-synuclein or Aβ42 and tau proteins, several similar neuronal pathophysiological cascades are triggered in PD and AD patients, leading to progressive neurodegeneration [11]. Several studies have reported increased α-synuclein aggregation in Lewy bodies in up to 50% of AD cases [12,13]. Statistical tests have not detected a significant difference in α-synuclein levels in cerebrospinal fluid between AD and PD patients [14]. In transgenic mice, α-synuclein accumulation can significantly impair cognitive function [13]. In a similar study, it was found that α-synuclein in transgenic mice could greatly increase apolipoprotein E (ApoE) levels and accelerate the accumulation of insoluble Aβ [15]. Total α-synuclein levels in cerebrospinal fluid have been shown to contribute to the differential diagnosis of AD and other dementias [16]. GWAS data were used to construct an interaction network, revealing a significant interaction between genes associated with AD and PD. Taken together, these findings suggest that the potential genetic mechanisms linking PD and AD-particularly the role of α-synuclein in AD-warrant further investigation. In recent years, large-scale studies have identified common genetic variants and provided further insights into the genetic architecture of AD and PD, including the gene encoding α-synuclein, *SNCA* [11].

The *SNCA* gene, located on chromosome 4q21-23, encodes α-synuclein, which modulates vesicle transport and neurotransmitter release at presynaptic terminals [17]. The association between α-synuclein and PD was first reported in 1997 when a missense mutation (A53T) in the *SNCA* gene was identified as cause of autosomal dominant parkinsonism [18]. Previous studies on *SNCA* gene polymorphisms have focused primarily on their association with PD [19]. It has been shown that triplication of the *SNCA* gene leads to dominant early-onset PD, suggesting that overexpression of wild-type α-synuclein may cause neurodegenerative disease [20,21]. Shortly afterward, interest in the link between *SNCA* gene polymorphisms and PD increased substantially [22,23]. At the same time, many researchers have demonstrated that significant accumulation of α-synuclein contributes to the pathogenesis of various neurodegenerative diseases such as PD, AD, multiple system atrophy (MSA), and Lewy body dementia (DLB) [24,25]. The presence of α-synuclein pathology in approximately 15–20% of clinically diagnosed AD cases suggests that genetic variations in the *SNCA* locus are potential risk factors [26].

Single nucleotide polymorphisms (SNPs) located in the *SNCA* locus can lead to biological consequences through alterations in gene expression, mRNA stability, regulatory region activity, or haplotype structures involving *SNCA* and neighboring genes. In this study, the rs2619363, rs2619364, rs2583988, and rs10005233 polymorphisms of the *SNCA* gene were investigated in patients with AD in the Turkish population and the association of these variants with AD was evaluated in a case–control study. The aim of the study was to determine the potential contribution of selected SNPs in the *SNCA* gene to the risk of AD in the Turkish population and to further elucidate the role of α-synuclein related mechanisms in the pathophysiology of AD at the genetic level.

## 2. Results

All enrolled registered participants completed the study procedures and were included in the analysis. Data from 95 patients with Alzheimer’s disease were compared with 97 control participants of similar age and gender. To validate the PCR-RFLP genotyping results, approximately 10% of the samples were randomly selected and subjected to independent restriction enzyme digestion, and the outcomes of the repeated genotyping revealed fully concordance with the initial calls, with no discordant genotypes identified.

The ages of the 192 participants ranged from 51 to 97 years, with a mean age of 75.67 ± 7.70. Of the participants, 65.6% were female. Regarding comorbidities, 38% had hypertension, 62% had diabetes mellitus, and 81.2% had coronary artery disease. Only 8.4% of the participants had a family history of AD. The demographic characteristics, medical history, genotype distribution, and allele frequencies of the participants are summarized in Table 1, with categorical variables expressed as counts and percentages. Firth regression, multi-locus genotype combinations, ordinal logistic regression, and Random Forest analyses were considered exploratory analyses and were not interpreted as confirmatory evidence.

The age and gender distribution of the participants did not differ significantly (*p* > 0.05) between the control and case groups. The rates of hypertension, diabetes mellitus, and coronary artery disease did not differ significantly (*p* > 0.05) between the control and case groups (Table 1). There were no significant differences (*p* > 0.05) in plasma glucose, HbA1c, TSH, triglycerides, HDL, WBC, neutrophils and PLT values between the control group and the case group. Total Cholesterol and LDL values were significantly lower in the case group than in the control group (*p* = 0.013, *p* < 0.001). Lymphocyte, RDW and MPV values were significantly lower in the case group than in the control group (*p* = 0.006, *p* < 0.001) (Table 1).

The presence of a family history of Alzheimer’s disease was not significantly different (*p* > 0.05) between the control and case groups. Although smoking was lower in the case group compared to the control group, there was no significant difference (*p* > 0.05) in smoking status between the control and case groups. Alcohol consumption was significantly lower in the case group compared to the control group (*p* = 0.006). The educational level was significantly lower in the case group compared to the control group (*p* < 0.001). The case group had a higher rate of illiteracy and primary school education, while the control group had a higher rate of higher education and university graduates (Table 1).

### 2.1. Genotype Distributions and Allele Frequencies

Significant differences (*p* < 0.05) were observed between the control and case group for the rs2583988, rs2619364, and rs2619363 polymorphisms.

The rs10005233 polymorphism did not show a significant difference (*p* > 0.05) between the control and case groups. In the case group, the CC genotype in the *SNCA* gene rs2583988 polymorphism was significantly more frequent compared to the control group, while the CT and TT genotypes were significantly less frequent compared to the control group (*p* < 0.001) (Table 2).

In the case group, the C allele in the *SNCA* gene rs2583988 polymorphism was significantly more frequent compared to the control group, while the T allele was significantly less frequent compared to the control group (*p* < 0.001). In the case group, the GG and GA genotypes in the *SNCA* gene rs2619364 polymorphism were significantly more frequent compared to the control group, while the AA genotype was significantly less frequent (*p* = 0.022) compared to the control group. In the case group, the G allele in the *SNCA* gene rs2619364 polymorphism was significantly more frequent compared to the control group, while the A allele was significantly less frequent (*p* = 0.007) compared to the control group (Table 2). In the case group, the GT and TT genotypes in the *SNCA* gene rs2619363 polymorphism were significantly more frequent than in the control group, while the GG genotype was significantly less frequent (*p* = 0.047) compared to the control group. Although the T allele was more frequent and the G allele was less frequent in the *SNCA* gene rs2619363 polymorphism in the case group compared to the control group, there was no significant difference (*p* = 0.054) between the case and control groups. No significant difference (*p* > 0.05) was found between the CC, CT, and TT genotypes in the *SNCA* gene rs10005233 polymorphism between the case and control groups. No significant difference (*p* > 0.05) was found between the C and T alleles in the *SNCA* gene rs10005233 polymorphism between the case and control groups (Table 2).

The analysis conducted using Firth penalized logistic regression revealed that the GT genotype was significantly correlated with an increased risk relative to the GG genotype (OR = 12.35, 95% CI: 1.37–1635.5, *p* = 0.021). On the other hand, the TT genotype did not demonstrate a significant association (*p* = 0.363). The overall model indicated borderline significance (likelihood ratio test, *p* = 0.051). The findings from the Firth penalized logistic regression analysis demonstrated that individuals with the AG genotype had a significantly heightened risk compared to those with the AA genotype (OR = 1.98, 95% CI: 1.07–3.72, *p* = 0.029). The GG genotype showed an increased odds ratio (OR = 4.88), although this did not reach statistical significance (*p* = 0.060). The overall model was statistically significant (likelihood ratio test, *p* = 0.025) (Table 3).

### 2.2. Univariate and Multivariate Logistic Regression Analysis of Factors Associated with Alzheimer’s Disease

Univariate logistic regression analysis revealed multiple genetic and clinical factors significantly associated with Alzheimer’s disease. Among the genetic variables, rs2583988 exhibited a robust protective association (OR = 0.421, *p* < 0.001), whereas rs2619363 (OR = 2.459, *p* = 0.030) and rs2619364 (OR = 1.439, *p* = 0.023) were associated with an increased risk.

In terms of clinical factors, higher mean platelet volume (MPV) was significantly associated with increased disease risk (OR = 1.686, *p* < 0.001), while red cell distribution width (RDW) (OR = 0.295, *p* < 0.001), low-density lipoprotein (LDL) (OR = 0.977, *p* < 0.001), and total cholesterol (OR = 0.991, *p* = 0.020) were inversely associated with disease presence. Additionally, thyroid-stimulating hormone (TSH) levels showed a significant negative association (OR = 0.723, *p* = 0.011). Alcohol consumption was also associated with a decreased risk (OR = 0.093, *p* = 0.025), while smoking demonstrated a borderline association (*p* = 0.075).

In the multivariate logistic regression analysis, only hematological and metabolic parameters were independently associated with Alzheimer’s disease. Red cell distribution width (RDW) showed a significant association with a decreased risk of the disease (OR = 0.211, *p* < 0.001), whereas mean platelet volume (MPV) was associated with an increased risk (OR = 1.803, *p* = 0.031). Additionally, low-density lipoprotein (LDL) levels demonstrated a modest but significant inverse relationship with disease status (OR = 0.971, *p* = 0.025). In univariate analyses, some *SNCA* variants were found to be associated with AD. However, in a multivariate logistic regression analysis that accounted for age, sex, education level, and clinical variables, *SNCA* variants did not remain independent predictors. In the multivariate model, only RDW, MPV, and LDL were found to be independently associated with AD. Therefore, findings related to *SNCA* variants should be interpreted as preliminary findings rather than independent genetic risk markers. Genetic variants (rs2619364, rs2619363, and rs2583988) as well as demographic factors (age, sex, and education) were not independently associated with the disease after adjustment for clinical parameters (Table 4).

### 2.3. Multi-Locus Genotype Combination Analysis and Linkage Disequilibrium of SNCA Polymorphisms

In the exploratory analysis of multi-locus genotype combinations, several genotype combinations showed nominal associations with Alzheimer’s disease. The counts of cases and controls for each combination are presented in Table 5. However, some combinations exhibited low frequencies, zero-cell counts, and wide confidence intervals. Therefore, these results should be interpreted with caution and considered exploratory rather than confirmatory.

**Figure 1:**
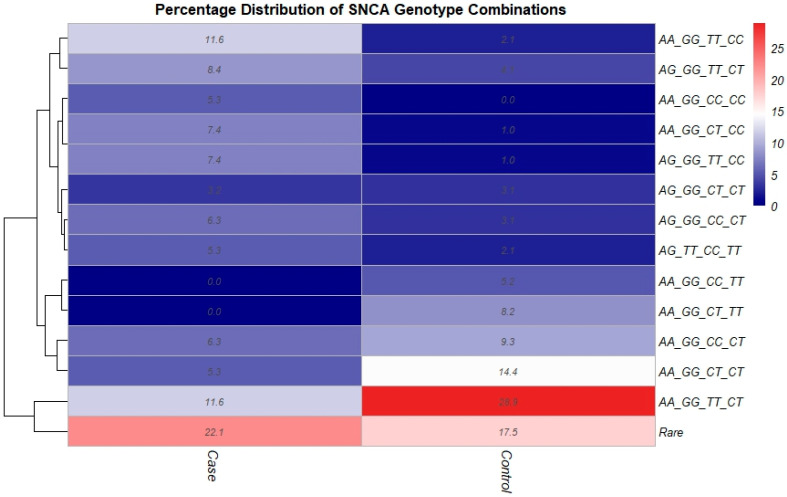
Heatmap showing the percentage distribution of *SNCA* genotype combinations in case and control groups.

The analysis of combined genotypes demonstrated that several patterns of the *SNCA* genotype were associated with an elevated risk of disease, with certain combinations showing significantly higher odds ratios. Moreover, heatmap visualization indicated that specific genotype combinations were enriched within the case group, while alternative combinations were more prevalent in the control group (Figure 1).

Pairwise linkage disequilibrium (LD) analysis conducted on the four *SNCA* polymorphisms indicated a generally weak LD across all pairs of SNPs. While some pairs of SNPs exhibited moderate D′ values, the r^2^ values were low (ranging from 0.007 to 0.050), which points to a limited correlation between the loci (Table 6).

### 2.4. Ordinal Logistic Regression Analysis of Cognitive Impairment Severity (Mini Mental State Examination (MMSE) Severity Categories)

This analysis using ordinal logistic regression demonstrated that none of the *SNCA* SNPs studied were significantly linked to the severity of cognitive impairment (all *p* > 0.05), suggesting that these genetic variants may be more associated with susceptibility to the disease rather than its progression or severity. Age and sex were also not significant predictors, although a non-significant trend indicating a higher risk in males was noted. On the other hand, education level exhibited a significant effect. Individuals with a primary school education had significantly lower odds of severe cognitive impairment (OR = 0.15, *p* = 0.028), and a similar trend was observed for those with a high school education, although it did not achieve statistical significance. In conclusion, these findings imply that while genetic variants may contribute to disease risk, sociodemographic factors such as education may have a more pronounced impact on clinical severity (Table 7).

### 2.5. Random Forest Analysis for Classification of Alzheimer’s Disease

The Random Forest analysis was conducted as an exploratory classification approach; it was not interpreted as evidence of clinical predictive value. Model performance was further evaluated by removing education from the model to analyze the potential influence of educational disparities between the case and control groups. Confusion matrices were developed for both models. The model demonstrated good classification performance, with rs2583988 emerging as the most important genetic predictor, followed by education level and rs2619364. A Random Forest model was established to differentiate Alzheimer’s patients from control individuals by employing genetic and clinical variables. Random Forest analysis was performed as an exploratory classification analysis. The model that included educational factors achieved an accuracy of 0.890, a sensitivity of 0.821, a specificity of 0.948, and an AUC of 0.974. When educational factors were removed from the model, the accuracy was 0.878, sensitivity was 0.810, specificity was 0.938, and AUC was 0.960. Thus, the exclusion of educational factors led to only a minimal decrease in model performance. However, due to the limited sample size and the lack of external validation, these results should be interpreted with caution and should not be taken as conclusive evidence of clinical predictive utility. The analysis of variable importance highlighted rs2583988 as the most critical predictor, followed by education level and rs2619364, while other variables such as age, sex, and rs2619363-rs10005233 contributed relatively less (Table 8). As multiple genetic comparisons were undertaken without the necessary formal correction for multiple testing, the *p*-values that have been reported should be viewed as nominal.

## 3. Discussion

This study investigates the effects of genetic variations in the *SNCA* gene, which encodes the alpha-synuclein protein, a key protein implicated in the pathogenesis of AD alongside amyloid-beta and tau. The data obtained in this study demonstrate that SNPs in the *SNCA* gene may play a critical role in understanding the heterogeneous nature of the disease and the processes of cognitive decline. SNPs in the alpha-synuclein (*SNCA*) gene have been studied extensively in PD and have been reported to be associated with the disease. This finding has been evaluated across different ethnic populations. However, the issue of *SNCA* polymorphisms predisposing to PD remains controversial when considering the study results. It is emphasized that the differences in genetic risk factors among ethnic populations may influence both disease and clinical presentation.

Aggregates of the alpha-synuclein protein, encoded by the *SNCA* gene, are the main component of Lewy bodies (LBs). Genetic polymorphisms and protein aggregations of *SNCA* gene have been shown to increase cognitive decline by interacting with amyloid-beta and tau proteins, thereby accelerating the toxic accumulation of amyloid plaques and neurofibrillary tangles in the brain. Focusing not only on amyloid plaques but also on synuclein pathology driven by *SNCA* genetic variation in Alzheimer’s disease may be useful in understanding the heterogeneous nature of the disease [27].

Genetic variability at the *SNCA* locus is generally transmitted via discrete blocks known as linkage disequilibrium (LD) blocks. Two main LD blocks have been proposed in the *SNCA* gene: the 5′ block extending from the promoter-enhancer region to exon 4, and the 3′ block containing intron 4, the 3′ untranslated region (UTR), and the 3′ end region of the gene [28,29]. SNPs in the 3′ block have shown a greater association with PD. The high number of markers in the 3′ block across different populations suggests a greater causal effect for variants located at the 3′ end compared to the 5′ end. The 3′ block in PD also contains elements with a higher conservation rate across species and may therefore have greater biological significance [30].

Among the various variants identified through GWASs and candidate gene analyses, the SNPs rs2583988, rs2619363, rs2619364, and rs10005233 form a critical genetic cluster located in the 5′ regulatory, proximal and intronic regions of the *SNCA* gene. While SNPs rs2583988, rs2619363, and rs2619364 are localized in the 5′ promoter (i.e., the region where transcription is initiated) and flanking regions, rs10005233 is located in intron 4. The intron 4 region is increasingly recognized for its role in alternative splicing regulation and the expression of non-coding antisense transcripts. Three SNPs-rs2583988, rs2619363, and rs2619364-located at the 5′ terminus of the *SNCA* gene have been extensively studied for their contribution to idiopathic PD and, more recently, AD pathology. These variants are situated in a region of the *SNCA* gene that exerts significant control over transcriptional activity. The 5′ regulatory SNPs (rs2583988, rs2619363, rs2619364) act as quantitative drivers of transcription and increase the availability of alpha-synuclein protein in critical cortical regions such as the temporal cortex [26,31]. This increased protein load facilitates the seeding of LBs and acts as a synergistic driver for the formation of amyloid beta accumulation. Amyloid-beta plaques and tau protein aggregation contribute to the formation of neurofibrillary tangles. Clinically, this manifests as the LB variant of Alzheimer’s disease (LBV/AD), which is characterized by more aggressive cognitive decline and increased neuropsychiatric symptoms [32]. The 5′ variants rs2583988, rs2619363, and rs2619364 show very high pairwise LD values, particularly in populations of European descent [33]. This high correlation suggests that these variants are frequently inherited together, forming a specific risk haplotype that can be used to predict disease susceptibility. The structural organization of these LD blocks varies considerably among ethnic groups [34].

Among the variants we examined, rs2583988 and rs2619363 are located in the 5′ LD block of the *SNCA* gene and are highly correlated with each other (r^2^ = 0.93) [31]. The literature suggests that these variants not only increase the risk of PD but also significantly increase the likelihood of comorbid LB pathology in AD cases. In particular, the carrier status of the rs2583988 risk allele has been associated with an increased risk of cognitive impairment in heterogeneous groups such as the Brazilian population, suggesting that this variant has a predominant effect on AD-like cognitive symptoms rather than motor symptoms. Furthermore, the fact that the effect of these variants becomes even more pronounced when interacting with the LRRK2 genotype highlights the importance of the complex genetic network underlying synucleinopathies [32]. In South American cohorts, particularly in Brazilian samples, although the rs2583988 risk allele T was found to be significantly more frequent in patients with cognitive impairment compared to control groups, in our study, the rs2583988 risk allele C was found to be significantly more frequent (*p* < 0.05) in the case group compared to the control group.

The heatmap displays distinct variations in the distribution of *SNCA* genotype combinations between the case and control groups. Certain combinations are more commonly found in cases, suggesting a potential role in disease susceptibility, while others are more prevalent in controls, indicating possible protective effects. The increased proportion of rare combinations in the case group further supports the idea that less common genetic profiles may contribute to disease risk. Additionally, the clustering patterns imply that specific genotype combinations may work together, emphasizing the importance of assessing combined genetic effects rather than individual SNPs alone.

Another important variant addressed in our study, rs10005233, is noteworthy for its strong association with REM sleep behavior disorder (RBD) and Lewy body dementia (DLB). Findings suggest that this variant is located in the intron 4 region of the *SNCA* gene and may lead to the production of truncated protein isoforms via alternative 3′ UTR usage point to a potential molecular mechanism underlying disease pathology. It is suggested that this variant may be present within a common haplotype with rs2583988, and that this shared genetic block may act as a driving force for synuclein accumulation across synucleinopathies. The rs10005233 variant occupies a unique position in the *SNCA* locus and serves as a critical marker for a spectrum of synucleinopathy that bridges the gap between prodromal sleep disorders and overt dementia. This variant, located in intron 4, is associated with different clinical endophenotypes independently, although it is in strong linkage disequilibrium with other 5′ region variants [35]. In our study, no significant association was found between rs10005233 and AD.

Alpha-synuclein’s contribution to AD is not merely additive; it exerts a synergistic effect. Protein overexpression is driven by variants such as rs2583988 and rs2619363, triggering a chain of molecular interactions that increase amyloid-beta and tau accumulation [31,36]. In addition, SNPs in the 5′ LD block, including the rs2619364 variant, have been observed to function as cis-regulatory elements involved in gene expression regulation. Specifically, haplotype 3, identified in the CT-rich region of intron 4, has been found to create a favorable molecular environment for LB development by increasing *SNCA* mRNA levels. The approximately 25% increase in *SNCA* mRNA detected in the temporal cortex of LBV/AD cases supports the hypothesis that alpha-synuclein overexpression is closely linked to AD pathology, accelerating neurodegeneration [26].

In this study, instead of limiting the *SNCA* locus to a single variant, we evaluated multiple SNPs together (rs2619363, rs2619364, rs2583988, rs10005233). In regions where linkage disequilibrium (LD) exists between closely located SNPs, the observed association may represent a specific haplotype structure or regulatory region rather than a single “causal” SNP. The significant association found in our study between rs2583988, rs2619364, and rs2619363 and AD in the *SNCA* gene may indicate a shared regulatory mechanism influencing *SNCA* expression.

The findings regarding the multi-locus genotype combinations must be approached with caution. While certain combinations exhibited nominal associations, many categories contained limited individual counts and broad confidence intervals. Additionally, linkage disequilibrium (LD) analysis revealed low r^2^ values among the SNPs examined, indicating that these loci are predominantly independent. Consequently, the results pertaining to genotype combinations should not be construed as indicative of a common risk haplotype, a robust LD block, or a clinically relevant screening marker.

The effect of variants in the *SNCA* gene on disease risk arises not from directly altering the protein sequence, but rather through modulation of gene expression and regulation. SNPs located in regulatory regions or intronic domains can alter α-synuclein levels by affecting processes such as transcription factor binding, chromatin accessibility, or alternative splicing. Increased α-synuclein levels or disruption of proteostasis balance may negatively impact synaptic function and may further accelerate aggregation processes through cross-seeding-like interactions with amyloid-β and tau. In this respect, the rs2583988 (C) and rs2619364 (G) risk alleles we identified in *SNCA* may increase *SNCA* expression or facilitate aggregation tendencies, thereby creating an additional pathway that exacerbates synaptic loss and neurotoxicity in AD. The lack of correlation between rs10005233 and AD suggests that this SNP has a distinct profile compared to other SNPs in the 5′ LD block.

The effect of *SNCA* can manifest not only in the risk it poses for the disease, but also in phenotypic characteristics such as age of onset, disease severity, pattern of cognitive domain involvement, or neuropsychiatric symptoms. Furthermore, variables such as gender, vascular risk factors, and environmental exposures can interact with *SNCA*, either enhancing or masking its effect. Therefore, reporting subgroup analyses may strengthen the biological interpretation of the findings. Genetic risks can be modified by environmental factors; researchers have observed that habits such as smoking, alcohol consumption, and cognitive activity may exert a protective effect against cognitive decline in genetically predisposed individuals [37]. The effect of *SNCA* has been studied in detail in this regard [38].

In our study, the groups were similar in terms of age, gender, comorbidities, medications used, and family history of Alzheimer’s disease. Although smoking rates were higher in the case group, no statistically significant difference was found. Alcohol use and education level were found to be significantly lower in the case group. The case and control groups differ in terms of certain demographic and clinical variables, particularly educational level. This limits the interpretation of univariate associations between *SNCA* variants and AD. The fact that genetic variants do not remain independent predictors in multivariate analysis suggests that these associations may be influenced by confounding variables. Therefore, *SNCA* variants should be interpreted not as strong independent risk markers, but as potential genetic associations that require validation in larger, well-matched studies. The proportion of participants who consumed alcohol was 5.7%, suggesting that alcohol use was relatively low. We obtained results consistent with the literature regarding alcohol and cigarette use [39,40].

The combined genotype evaluation suggests that various *SNCA* polymorphisms may exert a cumulative and potentially synergistic effect on disease susceptibility. While analyses of individual SNPs showed modest or genotype-specific influences, the multi-locus strategy revealed significantly higher odds ratios, indicating that genetic interactions among loci may play a crucial role. The identification of several high-risk combinations supports the hypothesis that *SNCA*-related disease risk is influenced by a complex genetic architecture rather than by single-variant effects. However, some genotype combinations exhibited extremely wide confidence intervals, reflecting limited sample size and sparse data in certain groups. Future studies with larger cohorts are needed to validate these results.

The literature on the association of *SNCA* variants with AD is not always consistent; the main reasons for these differences in sample size, ethnic background, diagnostic criteria, the extent of exclusion of comorbid LB pathology, and variability in gene-environment interactions across studies. Since α-synuclein pathology can be present even in cases that clinically appear as “pure Alzheimer’s,” differences in diagnostic classification can lead to heterogeneity in genetic association analyses. The findings of this study suggest that *SNCA* polymorphisms in the Turkish population may exert risk, protective, or neutral effect, and highlight potential differences in genetic background that may account for variability observed across populations. Our findings reflect this variability, with some SNPs showing risk effects, others protective effects, and neutral effects.

The considerable association observed solely in the heterozygous (GT) genotype, but absent in the homozygous variant (TT), may suggest a potential heterozygote effect. Nonetheless, the broad confidence intervals indicate limited statistical power and a possible imbalance in genotype distribution. The research findings propose that rs2619364 polymorphism may correlate with disease susceptibility, especially through the heterozygous AG genotype. Although the GG genotype showed a higher odds ratio, the absence of statistical significance and the wide confidence interval reflect limited statistical power, likely attributable to the small count of GG carriers. The increasing risk trend from AA to AG and GG may suggest a potential dose-dependent effect, which requires further investigation in more extensive cohorts.

The observed deviation from Hardy–Weinberg equilibrium (HWE) in certain SNPs within the control group is unlikely to be attributed solely to methodological errors. The study population was sourced from Istanbul, a highly cosmopolitan city characterized by significant internal migration, which may lead to population stratification (Wahlund effect) and contribute to the discrepancies in expected genotype distributions. Furthermore, the relatively high rates of consanguinity within the Turkish population may provide additional insight into the reduced heterozygosity noted at specific loci. It is also important to recognize that disease-associated variants may exhibit deviations from HWE in control groups due to a non-random distribution of genotypes associated with disease risk. Notably, the application of robust analytical methods, including penalized regression and machine learning techniques such as Random Forest, enhances the credibility of the findings despite these deviations. Consequently, the departures from HWE observed are more likely linked to population structure and biological influences rather than technical biases [41]. Education has been identified as one of the most critical variables in the Random Forest model; however, there was a pronounced imbalance in education levels between the case and control groups. Therefore, the model’s performance may, in part, be a reflection of the baseline differences between the groups, rather than the independent predictive contribution of *SNCA* variants.

Our study has some limitations. Firstly, the limited sample size may reduce the power to detect genetic associations with small effect sizes. Secondly, although only four SNPs were analyzed, multiple comparison corrections (e.g., Bonferroni/FDR) were not applied, and therefore the interpretation of statistically significant findings should be approached with caution. Thirdly, diagnosis was based on clinical criteria, and there is no single measure that fully demonstrates the effect of comorbid LB pathology. Finally, even if a genetic link is identified, complementary analyses of *SNCA* expression, α-synuclein levels, or relevant biomarkers may be required to establish functional relevance.

## 4. Materials and Methods

### 4.1. Patient Selection

**Figure 2:**
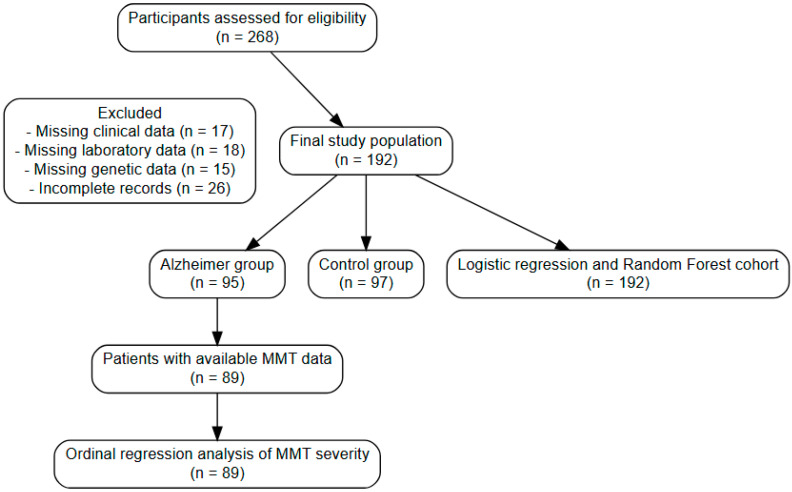
Study design and participant flow, including eligibility assessment, exclusions, and analytical cohorts.

The study included 95 patients with AD and 97 healthy controls. The patient group consisted of individuals diagnosed with Alzheimer’s Disease at the Neurology outpatient clinic of Istanbul Kanuni Sultan Suleyman Training and Research Hospital, Health Sciences University. The control group was selected from individuals without a history of cerebrovascular disease or neurological symptoms or diagnosis (Figure 2). MMSE scores were categorized as mild cognitive impairment (19–24), moderate cognitive impairment (10–18), and severe cognitive impairment (0–9), based on commonly used clinical cutoff ranges.

All participants underwent a detailed neurological examination, MMSE, and blood tests. Medical history, demographic data, and laboratory findings were recorded. A total of 5 mL of peripheral blood was collected from each participant into EDTA tube. Patients were recruited in an outpatient setting. Clinical examination, MMSE, and blood collection were performed on the same day. Selected SNPs in the *SNCA* gene, rs2583988, rs2619363, rs2619364, and rs10005233, were studied in all participants.

### 4.2. Genomic DNA Isolation

Approximately 5 mL of peripheral whole blood was obtained from dementia patients and age-matched controls into sterile tubes containing EDTA. Genomic DNA was extracted using Invitrogen PureLink Genomic DNA Mini Kit (Thermo Fisher Scientific, Waltham, MA, USA, Cat. No. K182002) in accordance with the manufacturer’s instructions. In summary, 200 µL of whole blood was placed into a sterile microcentrifuge tube, followed by the addition of 20 µL of Proteinase K (included in the kit, Cat. No. 25530-049) and 200 µL of Genomic Lysis/Binding Buffer (from the kit). The solution was briefly vortexed and incubated at 55 °C for 10 min to ensure complete lysis. Afterward, 200 µL of 100% ethanol (Thermo Fisher Scientific, Waltham, MA, USA; Cat. No. BP2818-500) was added and mixed thoroughly. The lysate was then transferred to a PureLink Spin Column placed in a collection tube and centrifuged at 10,000× *g* for 1 min; the flow-through was discarded. The column underwent sequential washing with 500 µL of Wash Buffer 1 and 500 µL of Wash Buffer 2 (with ethanol added as per instructions), each followed by centrifugation (1 min and 3 min, respectively, at 10,000× *g*). Following an optional dry spin to eliminate residual ethanol, DNA was eluted by adding 100 µL of Elution Buffer (10 mM Tris-HCl, pH 9.0; from the kit) directly onto the membrane, incubating for 1 min at room temperature, and centrifuging for 2 min at 10,000× *g*. The expected DNA yield was approximately 20–60 µg per 200 µL blood sample. DNA concentration and purity were assessed using a Tecan Infinite M1000 Pro microplate reader (Tecan Group Ltd., Männedorf, Switzerland) equipped with a NanoQuant Plate, and only samples exhibiting an A260/A280 ratio of 1.8 ± 0.1 were accepted. The integrity of the genomic DNA was confirmed through 1% agarose gel electrophoresis, stained with 3 µL of Safe-View Classic DNA Stain (Applied Biological Materials Inc., Richmond, BC, Canada; Cat. No. G108). The presence of a single high-molecular-weight band without smearing indicated that the DNA was intact and undegraded. All purified genomic DNA samples were stored at −20 °C until further analysis.

### 4.3. Genotyping of SNCA Polymorphisms

Genomic DNA samples previously extracted from peripheral blood were subjected to genotyping for the *SNCA* polymorphisms rs10005233, rs2619363, rs2583988, and rs2619364 using the polymerase chain reaction–restriction fragment length polymorphism (PCR-RFLP) technique. Specific primer pairs were synthesized by Probe Synthesis Biyoteknoloji A.S. (Ankara, Türkiye).

Each PCR amplification was conducted in a total reaction volume of 25 µL, which included 12.5 µL of MegaFi™ Pro Fidelity 2× PCR Master Mix (ABM Inc, Richmond, BC, Canada, Cat. No. G887), 0.5 µL of each primer (10 pmol), 1.5 µL of genomic DNA template, and 10.5 µL of nuclease-free water. The reactions were performed in a Techne Thermal Cycler (Bibby Scientific Ltd., Stone, UK) under the following cycling parameters: an initial denaturation at 95 °C for 5 min, followed by 35 cycles of denaturation at 95 °C for 45 s, primer-specific annealing for 30–45 s and a final extension at 72 °C for 45 s, and a concluding extension at 72 °C for 7 min. The PCR products were verified by 2% agarose gel electrophoresis prior to restriction digestion.

Restriction digestion was performed in a 15 µL total reaction volume that comprised 5 µL of PCR product, 0.4–0.5 µL of restriction enzyme (New England Biolabs, Ipswich, MA, USA), 1.5 µL of 10× NEBuffer, along with nuclease-free water. The mixtures were incubated at 37 °C for a period of 30–60 min, depending on the enzyme used. The resulting digested products were separated on 2.5–3.5% agarose gels, stained with Safe-View Classic DNA Stain (Abmgood; Applied Biological Materials Inc., Richmond, BC, Canada; Cat. No. G108) and visualized under UV light using a Minibis UV transilluminator (DNR Bio-Imaging Systems, Jerusalem, Israel).

The polymorphic site *SNCA* rs10005233 was amplified using the forward primer 5′-TGTCACTGTTCCTTTGGCAT-3′ and reverse primer 5′-AAGTCACTGTTATTCTACCACC-3′, with an annealing temperature of 55 °C. The size of the PCR product was 475 bp. Digestion was carried out using the restriction enzyme HaeIII (recognition site: GG↓CC; New England Biolabs, Ipswich, MA, USA; Cat. No. R0108). Samples containing the C allele yielded in two fragments measuring 265 bp and 210 bp, while the T allele remained intact, yielding a single fragment of 475 bp.

The *SNCA* rs2619363 polymorphism was amplified using the forward primer 5′-AAATCCTCTTTCCACGCCACT-3′ and reverse primer 5′-AAATTGGATATGATGGTCCGGTA-3′, at an annealing temperature of 65 °C. The PCR product size was 225 bp. Digestion was performed using the restriction enzyme AluI (recognition site: AG↓CT; New England Biolabs, Ipswich, MA, USA; Cat. No. R0137). The T allele yielded two fragments of 177 bp and 48 bp, whereas the G allele remained undigested at 225 bp.

At the *SNCA* rs2583988 locus, amplification was accomplished using the forward primer 5′-TGCCTCTTTACAACTAGTATCTCA-3′ and reverse primer 5′-TGCCACTTAAAATAGCTAGGTGA-3′, with an annealing temperature of 57.5 °C. The PCR product obtained was 388 bp length. Upon digestion with RsaI (recognition site: GT↓AC; New England Biolabs, Ipswich, MA, USA; Cat. No. R0167), the C allele yielded two fragments of 267 bp and 121 bp, while the T allele yielded three fragments of 192 bp, 121 bp, and 75 bp.

The *SNCA* rs2619364 variant was amplified using the forward primer 5′-AAAGCATGAAAGTAAGATCACAA-3′ and reverse primer 5′-CCTTTATTCTGTCTACTTTTGCAT-3′ at an annealing temperature of 51.3 °C. The PCR product was measured at 316 bp in length. Digestion was performed using the restriction enzyme HpaII (recognition site: C↓CGG; New England Biolabs, Ipswich, MA, USA; Cat. No. R0171). The A allele remained uncut, yielding a single 316 bp fragment, while the G allele was digested into two fragments of 188 bp and 128 bp.

The determination of genotypes was based on the specific banding patterns associated with homozygous wild-type, heterozygous, and homozygous variant genotypes at each locus. In each PCR run, both negative (no-template controls) and positive control DNA samples were included.

### 4.4. Haplotype Estimation and Linkage Disequilibrium Analysis

All statistical analyses were executed using R software (version 4.5.3; April 2026). For multi-SNP analyses, the SNPassoc package (version 2.3.1) was utilized, which incorporates archived functions from the haplo.stats package to facilitate haplotype inference. In the absence of directly observed linkage phase information, haplotype frequencies and individual posterior probabilities were estimated using the Expectation–Maximization (EM) algorithm. The association between the inferred haplotypes and the risk of Alzheimer’s disease (AH) was evaluated through logistic regression models within the generalized linear models (GLM) framework. Age and sex were included as covariates in the models. The most frequent haplotype was chosen as the reference category, and the results were presented as odds ratios (ORs) with 95% confidence intervals (CIs). Linkage disequilibrium (LD) for SNP pairs was analyzed through D, D′, and r^2^ statistics to quantify the strength and directionality of allelic association. All calculations were carried out in R, and the LD metrics for each SNP pair are summarized in Table 6. No graphical visualizations or haplotype network analyses were performed. A two-tailed *p*-value of <0.05 was considered statistically significant.

### 4.5. Genotype Combination Profiling and Heatmap Visualization

To analyze the distribution patterns of combined SNP genotypes, all possible multi-locus genotype combinations of the studied *SNCA* polymorphisms were generated for each individual. The frequency of each genotype combination was calculated separately for both the case and control groups and expressed as percentages. Genotype combinations with very low frequencies were classified under a ‘rare’ category to enhance interpretability and reduce sparsity. For visualization, a heatmap analysis was conducted to illustrate the percentage distribution of genotype combinations between the groups. Hierarchical clustering was employed to group similar genotype patterns according to their distribution profiles. The generation of the heatmap occurred in the R environment through the pheatmap package, with color intensity denoting the relative frequency of genotype combinations across the groups.

### 4.6. Random Forest Analysis

A Random Forest (RF) model was executed through the randomForest package in R to classify individuals as either Alzheimer’s disease cases or controls. The predictor variables consisted of *SNCA* polymorphisms (rs2583988, rs2619364, rs2619363, rs10005233) and demographic factors including age, sex, and education.

The model applied bootstrap resampling to generate an ensemble of decision trees, which improved predictive stability and minimized the likelihood of overfitting. The performance of the model was evaluated using accuracy (with 95% CI), sensitivity, specificity, balanced accuracy, Cohen’s kappa, and AUC.

The importance of features was measured using the mean decrease in accuracy alongside Gini impurity, yielding complementary evaluations of each variable’s impact on classification performance. All outcomes are summarized in Table 8. Given the limited sample size, an independent external validation cohort was not feasible. The results from the Random Forest were evaluated as exploratory findings, and no claims of clinical predictive utility were made.

### 4.7. Statistical Analysis

The primary hypothesis of this study is to assess whether the four selected *SNCA* variants are associated with susceptibility to Alzheimer’s disease. Basic SNP comparisons at the allele and genotype levels were considered the main genetic analyses. Firth regression, multi-locus genotype combinations, ordinal logistic regression, and Random Forest analyses were considered exploratory analyses. As these exploratory analyses did not undergo correction for multiple comparisons or external validation, they were viewed as generating hypotheses rather than serving as confirmatory evidence.

Descriptive statistics of the data included the mean, standard deviation, frequency, and percentage values. The distribution of variables was assessed using the Kolmogorov–Smirnov and Shapiro–Wilk tests. For normally distributed quantitative independent data, the independent samples *t*-test was used. For non-normally distributed quantitative independent data, the Mann–Whitney U test was used. For qualitative independent data, the chi-square test was applied, and when the assumptions of the chi-square test were not met, Fisher’s exact test was used. All analyses were performed using R software (version 4.5.3). The odds ratio (OR) is a quantifiable measure of association that reflects the strength of the relationship between a risk factor and a disease outcome. An OR that exceeds 1 denotes an increased risk, whereas an OR that falls below 1 indicates a protective effect.

An ordinal logistic regression analysis was executed to investigate the link between *SNCA* polymorphisms and the degree of cognitive impairment (MMSE severity categories), categorized into mild, moderate, and severe. This model included SNPs as predictors and was controlled for age, gender, and educational level.

Firth penalized logistic regression was employed to analyze the relationship between the selected SNP genotypes and the outcome. This method was specifically relevant for rs2619363 and rs2619364, owing to the sparse data and low-frequency genotype categories that might result in biased or unstable estimates in typical logistic regression models [42].

Univariate and multivariate logistic regression analyses were utilized to examine the associations between genetic and clinical variables and Alzheimer’s disease. Variables showing a *p*-value of less than 0.05 in the univariate analysis, along with clinically relevant factors, were integrated into the multivariate model to pinpoint independent predictors.

As a result of the study’s exploratory design and the restricted sample size, corrections such as Bonferroni or FDR were not implemented. Thus, nominal *p*-values are reported, and the genetic associations are viewed as exploratory insights instead of confirmatory data.

## 5. Conclusions

This study demonstrates a significant association between *SNCA* variants—particularly rs2583988, rs2619364, and rs2619363 and AD in the Turkish population—while rs10005233 was not significantly associated with AD. Even though some *SNCA* variants were correlated with AD in univariate analyses, they were not deemed independent predictors after adjusting for demographic and clinical factors. It also provides evidence that the rs2583988 (C) and rs2619364 (G) alleles in *SNCA* are associated with an increased risk of AD, whereas the rs2583988 (T) and rs2619364 (A) alleles in *SNCA* are associated with a reduced risk of AD. These SNPs may be involved in the genetic basis of susceptibility to Alzheimer’s disease (AD), but the independent predictive significance of these variants necessitates validation in larger and more adequately matched cohorts. The association of variants in the 5′ region, in particular, with cognitive components rather than motor function and with widespread neocortical LB distribution, suggests that these SNPs may serve as potential markers for further investigation in AD susceptibility. Despite some limitations, our findings reinforce the role of *SNCA* in AD pathogenesis and highlight the importance of large-scale, ethnically diverse studies to investigate gene-environment and gene-gene interactions, thereby supporting the development of personalized treatment strategies. Future studies may help to elucidate the biological mechanisms linking α-synuclein and AD. These findings may contribute to a better understanding of both shared and distinct pathological features of AD and PD and may encourage further research in this field. Examining the effects of these polymorphisms in different ethnic backgrounds, as well as their epigenetic regulatory mechanisms in larger cohorts, will further contribute to the development of personalized treatment strategies for AD. The Random Forest model was not validated externally, and the restricted sample size could have raised the risk of overfitting. Additionally, the significant influence of education, which showed notable differences between groups, restricts the interpretation of the model’s performance as evidence of genetic predictive utility.

Due to its geographical location, the Turkish population has served as a significant bridge between Asian, Middle Eastern, Caucasian, Balkan, and European populations, and possesses a complex structure characterized by a high degree of admixture and genetic heterogeneity. Large-scale whole-exome and whole-genome sequencing studies, such as the Turkish Variome (TR Variome) project, have detailed the genetic diversity of the Turkish population, its proximity to European populations, and the long runs of homozygosity it possesses due to its distinctive rates of inbreeding [43]. The genetic structure of Turkish population reveals high levels of variation and admixture.

## Figures and Tables

**Table 1:** Demographic, Clinical, and Laboratory Characteristics of the Study Groups.

Variable	Control (*n* = 97)	Case (*n* = 95)	*p*-Value
Age (years)	75.05 ± 5.39	76.21 ± 9.53	0.058
Gender (male)	37 (38.1%)	29 (30.5%)	0.266
Alcohol use (+)	10 (10.3%)	1 (1.1%)	0.006
Smoking (+)	17 (17.5%)	27 (28.4%)	0.073
Education level			<0.001
- No education	9 (9.3%)	10 (10.5%)	
- Primary school	53 (54.6%)	69 (72.6%)	
- Secondary school	6 (6.2%)	0 (0%)	
- High school	10 (10.3%)	5 (6.0%)	
- University	19 (19.6%)	0 (0%)	
Family history (+)	9 (9.3%)	7 (7.4%)	0.648
Hypertension (+)	64 (66.0%)	55 (57.9%)	0.249
Diabetes mellitus (+)	28 (28.9%)	27 (28.4%)	0.946
CAD (+)	17 (17.5%)	19 (20.0%)	0.661
Antihypertensive drug (+)	62 (63.9%)	55 (57.9%)	0.392
HbA1c (%)	6.33 ± 1.52	6.19 ± 1.06	0.298
FPG (mg/dL)	115.55 ± 43.20	113.44 ± 35.43	0.465
TSH (µIU/mL)	2.16 ± 1.43	1.67 ± 1.00	0.075
Total cholesterol (mg/dL)	208.9 ± 40.03	199.57 ± 55.60	0.013
Triglycerides (mg/dL)	156.25 ± 73.96	145.74 ± 82.42	0.067
LDL (mg/dL)	139.00 ± 32.20	115.83 ± 32.74	<0.001
HDL (mg/dL)	50.39 ± 11.88	47.09 ± 12.44	0.156
WBC (×10^9^/L)	8.56 ± 5.98	8.10 ± 4.37	0.155
Neutrophils (×10^9^/L)	4.51 ± 1.89	4.59 ± 1.64	0.378
Lymphocytes (×10^9^/L)	4.40 ± 8.65	1.96 ± 0.81	0.006
Platelets (PLT, ×10^9^/L)	251.90 ± 76.88	230.03 ± 60.03	0.137
RDW (%)	16.55 ± 0.79	14.20 ± 1.66	<0.001
MPV (fL)	9.41 ± 1.31	10.26 ± 1.25	<0.001

Values are expressed as mean ± SD (median) or *n* (%).

**Table 2:** Genotype and Allele Distributions of *SNCA* Polymorphisms in Case and Control Groups.

SNP	Genotype/Allele Frequency	Control *n* = 97 (%)	Case *n* = 95 (%)	*p*-Value	OR (95% CI)	HWE (*p*-Value) in Controls
rs2583988	CC	5 (5.2%)	43 (45.3%)	<0.001 ^c^	1.00 (Ref)	<0.001
	CT	63 (64.9%)	45 (47.4%)		0.08 (0.03–0.23)	
	TT	29 (29.9%)	7 (7.4%)		0.03 (0.01–0.10)	
	T	121 (62.4%)	59 (31.1%)	<0.001	1.00 (Ref)	
	C	73 (37.6%)	131 (68.9%)		3.68 (2.41–5.61)	
rs2619364	AA	72 (74.2%)	54 (56.8%)	0.022 ^c^	1.00 (Ref)	0.47
	AG	24 (24.7%)	36 (37.9%)		2.00 (1.08–3.70)	
	GG	1 (1.0%)	5 (5.3%)		6.66 (0.76–58.4)	
	A	168 (86.6%)	144 (75.8%)	0.007 ^c^	1.00 (Ref)	
	G	26 (13.4%)	46 (24.2%)		2.06 (1.21–3.51)	
rs2619363	GG	90 (92.8%)	80 (84.2%)	0.047 ^c^	1.00 (Ref)	<0.001
	GT	0 (0%)	5 (5.3%)		N/A	
	TT	7 (7.2%)	10 (10.5%)		1.60 (0.57–4.49)	
	G	180 (92.8%)	165 (86.8%)	0.054 ^c^	1.00 (Ref)	
	T	14 (7.2%)	25 (13.2%)		1.94 (0.98–3.84)	
rs10005233	CC	25 (25.8%)	30 (31.6%)	0.442 ^c^	1.00 (Ref)	0.005
	CT	31 (32.0%)	23 (24.2%)		1.62 (0.79–3.32)	
	TT	41 (42.3%)	42 (44.3%)		1.17 (0.60–2.30)	
	C	81 (41.8%)	83 (43.7%)	0.702 ^c^	1.00 (Ref)	
	T	113 (58.2%)	107 (56.3%)		1.08 (0.72–1.62)	

^c^ *p*-values were calculated using Pearson’s chi-square test.

**Table 3:** Associations Between rs2619363 and rs2619364 Genotypes and Outcome: Results from Firth Penalized Logistic Regression.

SNP	Genotype	Firth OR (95% CI)	Firth *p*-Value	SNP	Genotype	Firth OR (95% CI)	Firth *p*-Value
rs2619363	GG	1.00 (Ref)		rs2619364	AA	1.00 (Ref)	
	GT	12.35 (1.37–1635.5)	0.021		AG	1.98 (1.07–3.72)	0.029
	TT	1.57 (0.59–4.37)	0.363		GG	4.88 (0.94–48.56)	0.060

**Table 4:** Univariate and Multivariate Logistic Regression Analysis of *SNCA* Polymorphisms and Clinical Variables Associated with Alzheimer’s Disease.

Variable	Univariate OR (95% CI)	*p*-Value	Multivariate OR (95% CI)	*p*-Value
rs2583988	0.421 (0.286–0.605)	<0.001	--	--
LDL	0.977 (0.967–0.987)	<0.001	0.971 (0.943–0.994)	0.025
RDW	0.295 (0.209–0.396)	<0.001	0.211 (0.104–0.359)	<0.001
MPV	1.686 (1.331–2.181)	<0.001	1.803 (1.088–3.219)	0.031
TSH	0.723 (0.557–0.923)	0.011	--	--
Total Cholesterol	0.991 (0.984–0.998)	0.020	--	--
rs2619364	1.439 (1.055–1.978)	0.023	--	--
Alcohol Use	0.093 (0.005–0.498)	0.025	--	--
rs2619363	2.459 (1.159–6.071)	0.030	--	--
Smoking	1.869 (0.947–3.772)	0.075	--	--

Variables included in the multivariate model were selected based on univariate significance and clinical relevance. “--” indicates variables not retained in the final multivariate model.

**Table 5:** Exploratory Association Analysis of Multi-Locus Genotype Combinations of *SNCA* Polymorphisms with Alzheimer’s Disease.

Genotype Combination	Control *n* (%)	Case *n* (%)	OR (95% CI)	*p*-Value
AA_GG_TT_CT	28 (28.9%)	11 (11.6%)	0.32 (0.14–0.73)	0.004
AA_GG_CT_TT	8 (8.2%)	0 (0%)	0 (0–0.57)	0.007
AA_GG_TT_CC	2 (2.1%)	11 (11.6%)	6.17 (1.29–58.77)	0.010
AA_GG_CC_CC	0 (0%)	5 (5.3%)	Inf (0.96–Inf)	0.028
AA_GG_CT_CC	1 (1.0%)	7 (7.4%)	7.57 (0.94–347.10)	0.034
AG_GG_TT_CC	1 (1.0%)	7 (7.4%)	7.57 (0.94–347.10)	0.034
AA_GG_CT_CT	14 (14.4%)	5 (5.3%)	0.33 (0.09–1.03)	0.051
AG_GG_CC_CC	0 (0%)	4 (4.2%)	Inf (0.68–Inf)	0.058
AA_GG_CC_TT	5 (5.2%)	0 (0%)	0 (0–1.09)	0.059
AA_TT_CT_CC	0 (0%)	3 (3.2%)	Inf (0.42–Inf)	0.119
AG_GG_CT_TT	4 (4.1%)	0 (0%)	0 (0–1.53)	0.121
AG_GG_CC_TT	3 (3.1%)	0 (0%)	0 (0–2.46)	0.246
AG_GG_TT_CT	4 (4.1%)	8 (8.4%)	2.13 (0.55–10.02)	0.248
AG_TT_CC_TT	2 (2.1%)	5 (5.3%)	2.63 (0.42–28.24)	0.276
AG_GG_CC_CT	3 (3.1%)	6 (6.3%)	2.10 (0.43–13.40)	0.328
**Rare combinations**	7 (7.2%)	11 (11.6%)	1.68 (0.56–5.36)	0.332
AA_GG_CC_CT	9 (9.3%)	6 (6.3%)	0.66 (0.19–2.18)	0.592
AG_GG_CT_CC	1 (1.0%)	2 (2.1%)	2.06 (0.11–122.90)	0.619
AG_GG_CT_CT	3 (3.1%)	3 (3.2%)	1.02 (0.13–7.83)	1.000
AG_GG_TT_TT	2 (2.1%)	1 (1.1%)	0.51 (0.01–9.89)	1.000

The calculation of odds ratios (ORs) was performed for each genotype combination relative to all other combinations. Rare combinations were consolidated because of their low frequency. Owing to the low counts and wide confidence intervals, the results ought to be interpreted as exploratory; *p*-values are nominal.

**Table 6:** Linkage Disequilibrium (LD) analysis among *SNCA* polymorphisms.

SNP Pair	D	D′	r^2^
rs2619364–rs2619363	0.012	0.148	0.011
rs2619364–rs10005233	−0.019	0.179	0.010
rs2619364–rs2583988	0.016	0.161	0.007
rs2619363–rs10005233	−0.033	0.574	0.050
rs2619363–rs2583988	0.023	0.432	0.024
rs10005233–rs2583988	−0.038	0.170	0.024

**Table 7:** Ordinal Logistic Regression Analysis of Factors Associated with Cognitive Impairment Severity Based on MMSE categories.

Variable	β (SE)	OR (95% CI)	*p*-Value
**SNPs**			
rs2619364	0.100 (0.243)	1.11 (0.69–1.78)	0.681
rs2619363	−0.428 (0.474)	0.65 (0.26–1.65)	0.367
rs10005233	−0.163 (0.282)	0.85 (0.49–1.48)	0.563
rs2583988	−0.102 (0.241)	0.90 (0.56–1.45)	0.672
**Covariates**			
Age	0.007 (0.028)	1.01 (0.95–1.06)	0.811
Sex (Male vs. Female)	0.789 (0.500)	2.20 (0.83–5.86)	0.114
Primary School	−1.884 (0.859)	0.15 (0.03–0.82)	0.028
High School	−2.636 (1.424)	0.07 (0.00–1.17)	0.064

The model was adjusted for age, sex, and education level. MMSE severity categories were categorized as mild, moderate and severe cognitive impairment.

**Table 8:** Exploratory Random Forest Model Performance, Variable Importance, and Confusion Matrix for Classifying Alzheimer’s Disease.

Panel A. Model Performance	Value			Panel B. Variable Importance	Mean Decrease Accuracy	Mean Decrease Gini
	**Original Model**	**Education Included**	**Education Excluded**			
Accuracy	0.796	0.89	0.878	rs2583988	27.51	10.58
95% CI	0.665–0.894	--	--	Education	21.64	8.41
Sensitivity	0.760	0.821	0.810	rs2619364	17.33	5.79
Specificity	0.828	0.948	0.938	Age	7.93	13.02
Balanced Accuracy	0.794	--	--	rs2619363	10.79	2.62
Kappa	0.589	--	--	Sex	4.49	2.43
AUC	0.819	0.974	0.96	rs10005233	−3.21	3.41
**Model (Confusion matrix)**	**Prediction**		**Actual Control**		**Actual AD**	
Education included	Predicted Control		92		15	
	Predicted AD		5		69	
Education excluded	Predicted Control		91		16	
	Predicted AD		6		68	

## Data Availability

The data that support the findings of this study are available from the corresponding author upon reasonable request.
